# Calcifying Aponeurotic Fibroma: A Multimodal Imaging Description of an Unusual Case Involving Soft Tissues Adjacent to the Clavicle

**DOI:** 10.1016/j.radcr.2022.02.001

**Published:** 2022-03-02

**Authors:** Javier Sanmiguel, Pablo Santiago Diaz, Salvatore Marsico, José Lloreta Trull, Alberto Solano López

**Affiliations:** a Department of Radiology, Hospital Universitario Lucus Augusti. Rúa Dr. Ulises Romero, 1, 27003 Lugo, España; b Department of Pathological Anatomy, Hospital del Mar, Passeig Marítim de la Barceloneta, 25, 29, 08003 Barcelona, España; c Department of Radiology, Hospital del Mar, Passeig Marítim de la Barceloneta, 25, 29, 08003 Barcelona, España

**Keywords:** Clavicle, MRI, CAF

## Abstract

Calcifying aponeurotic fibromas (CAFs) are rare benign tumors that typically develop in the soft tissue of the extremities. We report a case of 64-year-old woman with a CAF in the soft tissue surrounding her left clavicle. A plain radiograph showed an asymmetrical increase in opacity of the left internal clavicular region. Computed tomography and magnetic resonance imaging confirmed the presence of a heterogeneous lesion of the periclavicular soft tissue, with peripheral calcifications, and remodeling of the adjacent clavicular bone. Following ultrasound-guided biopsy and surgical resection of the mass, the final histological diagnosis was made. To the best of our knowledge, this is the first case of a CAF described in the soft tissue adjacent to the clavicle. It is essential to use all the diagnostic methods available (X-ray, ultrasound, CT, MRI, and percutaneous biopsy) to obtain the final diagnosis of this rare disease.

## Introduction

In 1953, Keasbey first described a soft tissue lesion located in the hands and feet of four children; he classified the lesion as "juvenile aponeurotic fibroma." In 1964, Lichtenstein and Goldman termed it "fibromatosis cartilage analog." In 1973, Iwasaki and Enjoji termed the same lesion “calcifying aponeurotic fibroma” (CAF) after it was found that it was present not only in children but also in adolescents and adults [[1], [2], [3], [4], [5],^7^,^11^,^15^]. Since its initial discovery, more than 150 cases have been documented in the literature [12]. Most cases occur in the hands [10], fingers [6,9], and plantar aspect of the feet [3,12], but they can also arise from the connective tissues of the neck, abdominal wall, back, knee [8,11], thigh, arm, forearm, and elbow [5]. The lesion has also been detected surrounding a joint or invading the adjacent joint, simulating gout or calcium pyrophosphate deposition disease [14]. More than 50% of recurring lesions do so locally and, in these cases, more frequently in patients younger than 5 years of age, typically within the first 3 years postoperatively. Furthermore, there is no clear evidence of familial or ethnic association [2,3]. We present a unique case of a CAF with an atypical localization iin the soft tissue adjacent to the left clavicle. Although well known, CAF can pose a diagnostic challenge, particularly when it occurs at an older age and in atypical locations, as in our case. CAFs present characteristic imaging features that may mimic malignant lesions; therefore, it should always be considered in the differential diagnosis of chondroid and sarcomatous soft tissue lesions.

## Case report

**Fig 1:**
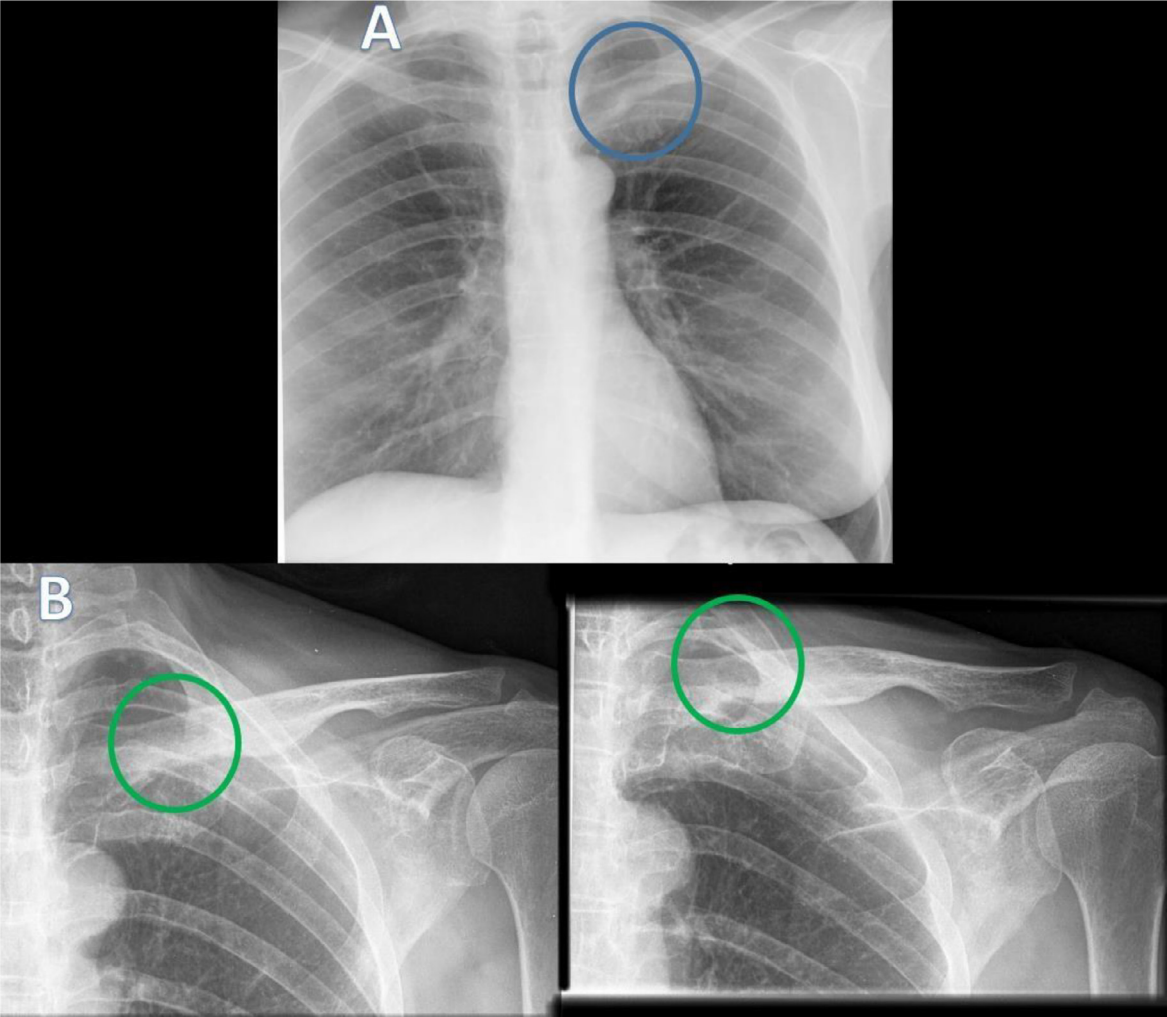
Standard chest radiograph in the posterior-anterior view (A) and plain X-ray film of the left clavicle (B) showing increased opacification at the left medial clavicular region (blue circle). The plain X-ray film of the left clavicle shows concentric thickening of the medial third and middle third of the left clavicle without involving the cortex (green circles).

A 64-year-old female patient was referred to our hospital with a history of long-term polyarthralgia and myalgia. She also presented with swelling of the left clavicular region that had been present for 3 years and had progressively increased in size and become more painful. On physical examination, there were no motor or sensory function abnormalities in the left upper limb. Routine laboratory tests showed no significant alterations. A standard chest radiograph revealed clear asymmetry and increased opacification at the left medial clavicular region (Fig. 1A). Plain radiograph of the left clavicle showed a concentric thickening of the medial third and middle third of the left clavicle without involvement of the cortex (Fig. 1B).

No-contrast computed tomography (CT) revealed the presence of a soft tissue lesion with chondroid calcifications located under the left pectoral muscles: between the first rib, its chondrocostal joint, and the clavicle (Fig. 3). It was associated with new bone formation and erosions of the inferior clavicular margin. The size of the lesion was approximately 49 × 28 × 21 mm. Magnetic resonance imaging (MRI) of the left clavicle was then performed, which confirmed a heterogeneous soft tissue lesion located adjacent to the distal end of the left clavicle with the following signal characteristics: (1) In T1-weighted sequences, the lesion was predominantly isointense or slightly hyperintense, relative to adjacent muscles; (2) in the T2-weighted and T2FS-weighted sequences, the lesion appeared hyperintense but had a predominantly hypointense marginal portion due to the presence of calcifications. The distal end of the clavicle was extensively eroded and displayed significant contrast enhancement in both the soft-tissue lesion and affected clavicle bone. A poor cleavage plane was found between the lesion and the adjacent subclavian neurovascular bundle.

Because of these findings, the initial diagnostic consideration was synovial osteochondromatosis, although it was not possible to completely rule out a primary cartilaginous tumor of the soft tissue, such as chondroma, periosteal chondroma, or chondrosarcoma. Following an ultrasound B-Mode evaluation (Fig. 5), an ultrasound-guided biopsy of the lesion was performed using a Tru-cut biopsy needle (16 Gauge × 10 mm), from which two cylinders of tissue were obtained for pathological evaluation. Macroscopically, these two pieces of tissue had a rough surface, whitish color, and elastic consistency. On light microscopy, the sections were found to consist of alternating areas of chondromyxoid and dense connective tissue, both with relatively scarce cells. No significant atypia was observed (Figs. 6 and 7). There was no necrosis or hypercellularity and the lesion did not appear to infiltrate adjacent tissues. The microscopic features differed from those of a typical synovial chondromatosis or a sarcomatous lesion. Instead, the special distribution of calcification in the center of the chondroid metaplasia surrounded by a denser connective tissue was highly reminiscent of a CAF. To confirm that this morphologic pattern was only present in the tumor, and considering that the tumor was very painful, a wide surgical excision was performed. The surgical specimen measured 70 × 35 × 28 mm, and it had an irregular external surface with obvious muscle and fat tissue (Fig. 7). On the cut surface, the mass was ovoid, nonencapsulated, and poorly demarcated from the adjacent soft tissues but without infiltrative areas, and measured 30 × 20 × 12 mm. A zonal pattern was grossly apparent with an outer soft area and a pearly-white central portion in which a mottled pattern was identified with the aid of a magnifying dissecting microscope.

Microscopic examination revealed a multinodular, lobulated, architectural pattern with extensive areas of calcified cartilaginous tissue, adjacent non-calcified cartilage remnants, and an outer fibroblastic component without atypia, necrosis, or mitoses (Fig. 8). Most of the tumor was surrounded by skeletal muscle and small areas of the lesion were obviously in contact with the surgical margins. The previously stated microscopic appearance was suggestive of a CAF, with the particularity of an unusual amount of calcium deposition in the cartilaginous component.

## Discussion

CAFs are benign fibroblastic tumors located in the subcutaneous tissue [7]. CAFs generally occur in the first or second decade of life, although cases have been reported in patients up to 64 years of age. The mean age at diagnosis is 12 years [1,2,6]. In our case, the patient was 64 years old, in the upper end of the age range described in previous studies. The tumor was slow growing, presenting as a firm mass, and its limits were relatively defined, firm on palpation. It was painless (although painful lesions have also been reported). Except for tumors that arise outside of the hands and feet, CAFs are usually not very large (rarely more than 3 cm in diameter) [2,5]. Our case presented several features typical of CAFs; it was initially a very slow growing mass, which had been present for 3 years, and resulted in a progressive increase in pain. To the best of our knowledge, this is the first case of a CAF reported in this thoracic location. As previously mentioned, CAF lesions are benign, but have a high incidence of nondestructive recurrence after surgical excision. Due to these characteristics, CAFs can be underdiagnosed, underreported, or even misdiagnosed, leading to unnecessary amputations [7].

Enzinger and Weiss suggested that CAFs develop biphasically, with initial and late phases. In the initial phase, seen more frequently in young patients, the tumor has an infiltrative and destructive growth and often lacks calcification; while in the late phase the tumor is more compact and nodular and exhibits a more prominent degree of calcification and cartilage formation [4,5,8,13,18]. The standard of care for CAFs includes proper diagnosis and conservative surgical treatment. Therefore, imaging appearances of CAFs may vary depending on the age of the patient, presence of calcifications, and bone involvement [6]. Its molecular basis remains unknown. The FN1-EGF fusion, which has not been observed in any other neoplasm, appears to be the main driver of mutation in CAFs. More functional studies are required to understand the exact pathogenesis of CAF [2]. Radiographic features are also not pathognomonic [2]. A nonspecific soft-tissue mass may be seen with or without the presence of fine punctate calcifications [6]. Therefore, the calcification seen in the pathological findings may not always be evident on a normal radiograph and there may not always be a correlation between the appearance or pattern of the calcification on the radiograph and the pathological calcification. Signs of bone involvement such as extrinsic erosion and thickening of adjacent bone may be present [17].

**Fig 2:**
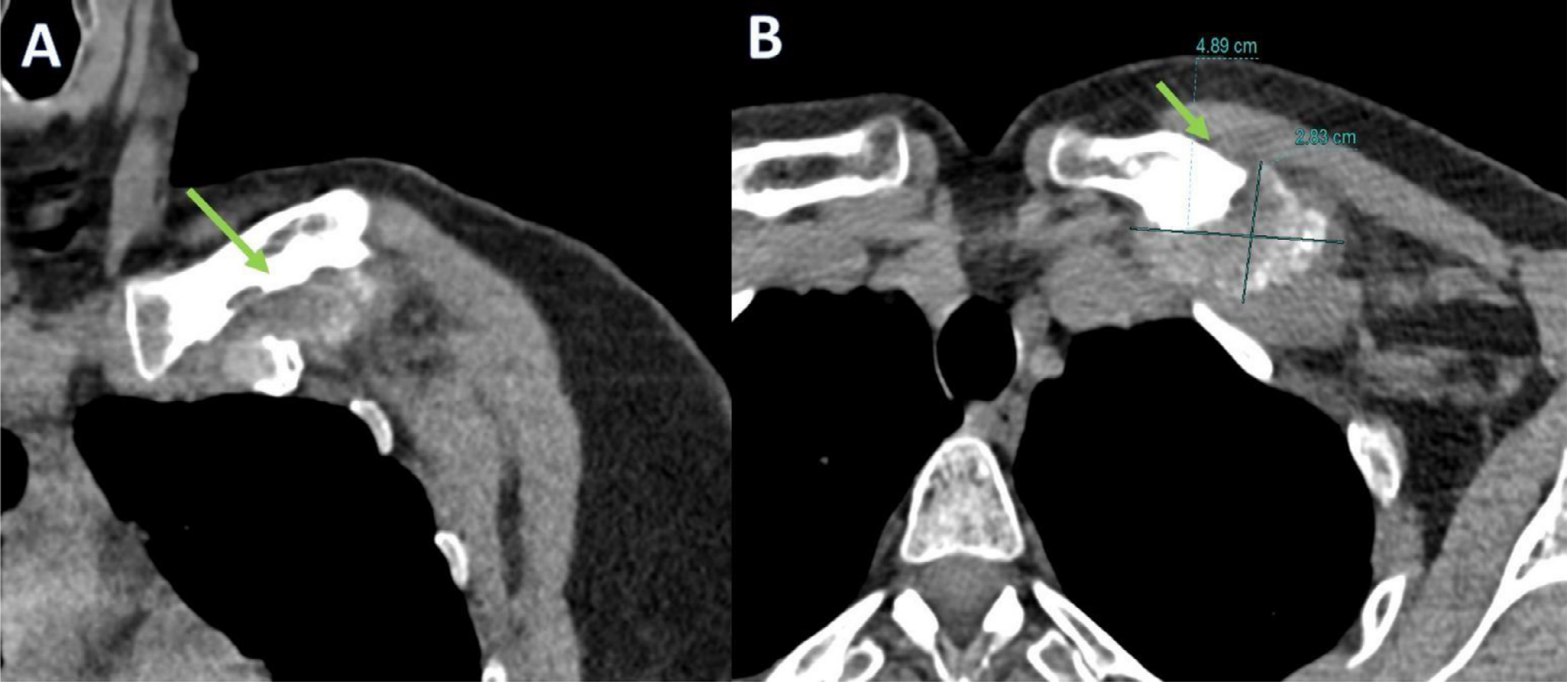
Volumetric computed tomography of the left upper thoracic area in the coronal (A) and axial (B) planes detecting soft tissue lesion with chondroid calcifications, between the 1st rib, its chondrocostal joint, and the clavicle associated with new bone formation and erosions of the inferior clavicular margin (green arrows).

MRI and CT are the imaging modalities of choice for CAFs [4]. CT is useful to determine calcified areas in the lesion and its association with the adjacent bone [6]. CT usually reveals a nonspecific soft tissue mass with stippling of calcification, in addition to showing the infiltrative growth pattern of the lesion in the surrounding tissues [4,[13], [14], [15], [16],^19^]. On MRI, CAF often appears as a poorly defined subcutaneous mass with intermediate to low signal intensity on T1-weighted sequences [6]. Low to intermediate intensity or isointensity on T1-weighted images can be attributed to the fibrous component, degree of cellularity, and presence of calcification [2]. However, on T2-weighted images, some authors demonstrated that CAFs exhibited low signal intensity, while others reported that CAFs exhibited homogeneous or heterogeneously high signal intensity. This variability in signal intensity on the T2-weighted images is likely dependent on the degree of hypocellularity or the amount of calcification or collagen within the tumor. Therefore, the calcification, the fibrous component, and the cellularity influence the characteristics of the T2-weighted signal [3,11,14,15,19]. Prominent areas of low globular signal intensity can be seen in all MRI pulse sequences, corresponding to the presence of calcification. CAF generally shows intense heterogeneous enhancement after intravenous administration of gadolinium [6]. Definitive diagnosis of CAF is based on histological findings [2]. CAFs are composed of fibroblasts with round and ovoid nuclei. There are foci of calcification and scattered chondroid areas surrounded by proliferating fibroblast protrusions with round or oval nuclei. Mitotic activity is rare. In some cases, osteoclast- like multinuclear giant cells are also present around the calcified area. However, ossification is rare [2,4,5,13,14,19]. Our case showed some diagnostic criteria typical of CAF. First, it shows some typical clinical features (painful, slow growth). In our case, conventional radiography did not significantly contribute to the diagnosis as it showed concentric thickening of the medial and middle thirds of the left clavicle with apparent preservation of the cortical bone. CT and MRI contributed more to the diagnosis because chondroid calcifications, located under the pectoral musculature, between the 1st rib, its chondro-costal joint, and the clavicle, were identified in CT. MRI showed a pattern similar to previous cases of CAF, with an appearance that was predominantly iso-hypointense in T1-weighted sequences and hyperintense in T2-weighted sequences, with a calcification pattern in its most marginal portion and poor cleavage plane with neighboring structures (Figs. 2, 4).

**Fig 3:**
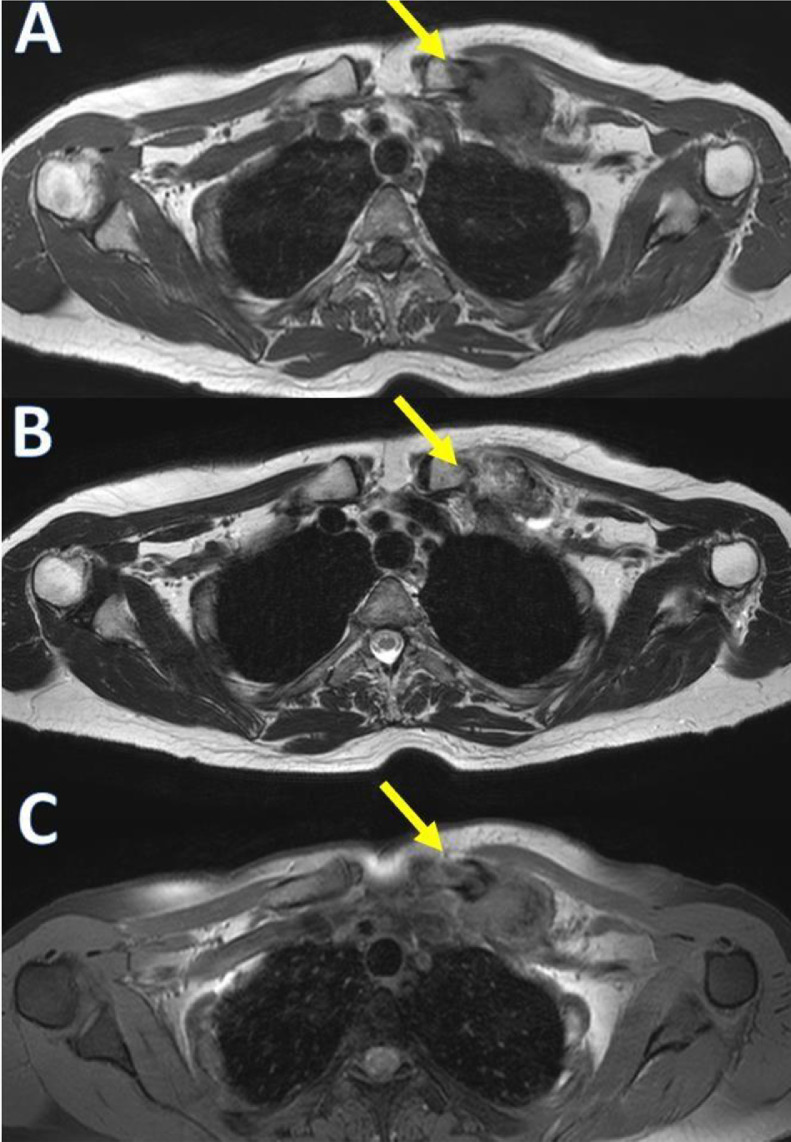
Magnetic resonance imaging (MRI) using T1 (A), T2 (B), and T1 Fat Sat (C) weighted sequences in the axial plane indicate a soft tissue lesion predominantly isointense or slightly hyperintense, relative to adjacent muscles in T1-weighted sequence and predominantly hyperintense in T2-weighted sequences (yellow arrows). Hypointense marginal portion, due to the presence of calcifications, is observed in both T1 and T2 weighted images (green arrows).

**Fig 4:**
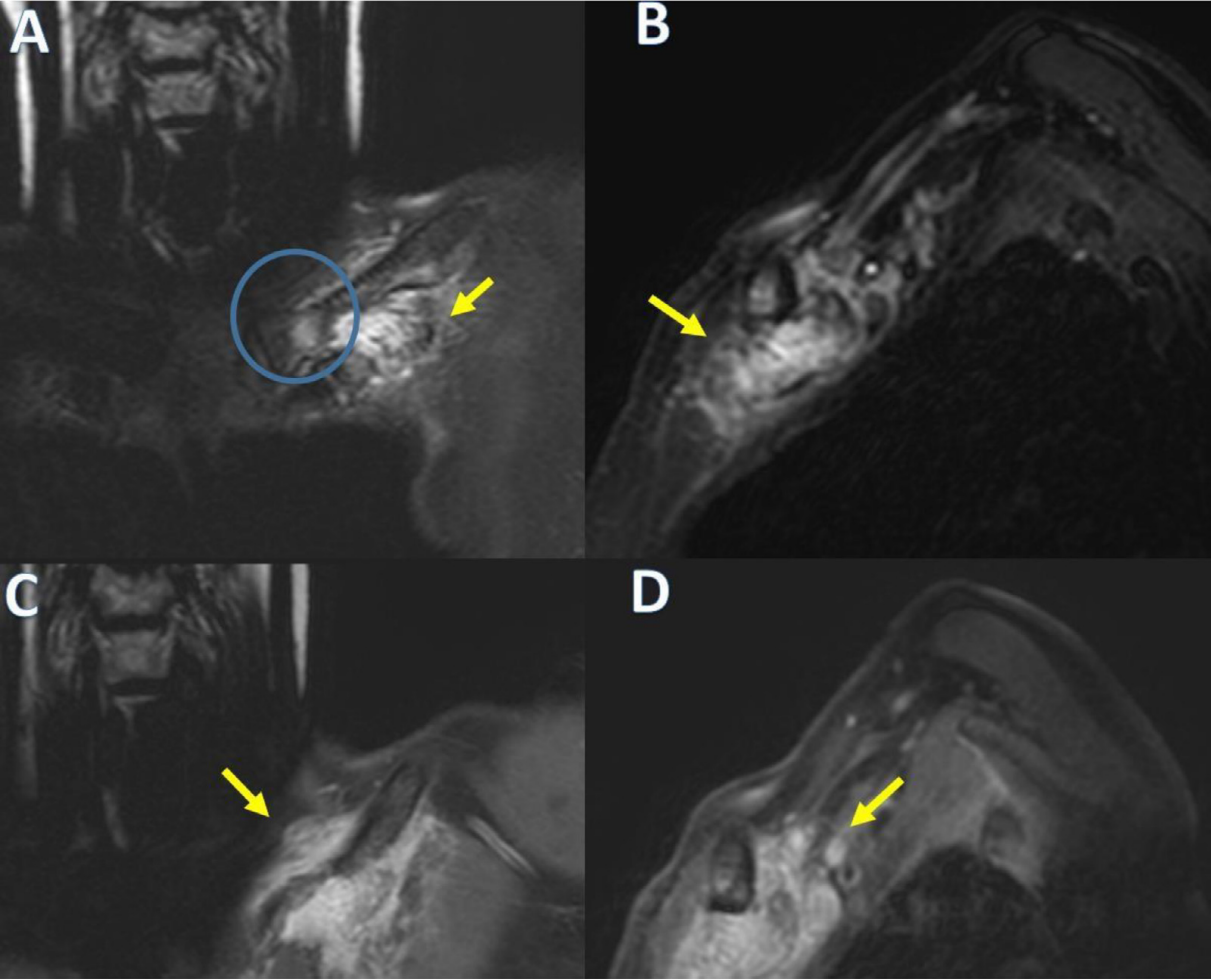
Magnetic resonance imaging (MRI) using T2 Fat Sat (A in sagittal plane and B in coronal plane), and post contrast T1 Fat Sat (C in sagittal plane and D in coronal plane) weighted sequences show a soft tissue mass predominantly hyperintense in T2 FatSat-weighted sequences (yellow arrows) with extensive and diffuse contrast enhancement that extends to the adjacent soft tissue. Mild edema and post contrast enhancement of the adjacent clavicle (green arrow) is also observed.

**Fig 5:**
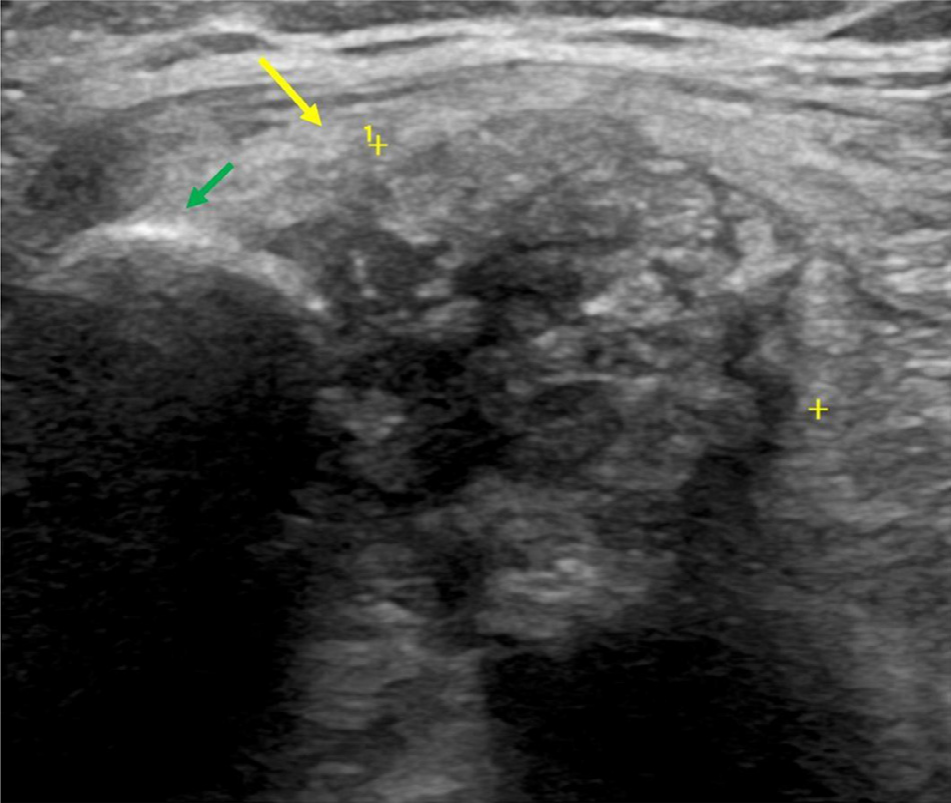
B-Mode ultrasound image in the transverse plane which confirms the presence of an ovoid mass containing hyperechoic images within, suggestive of calcifications (yellow arrow) in close contact with the clavicular bone (green arrow).

**Fig 6:**
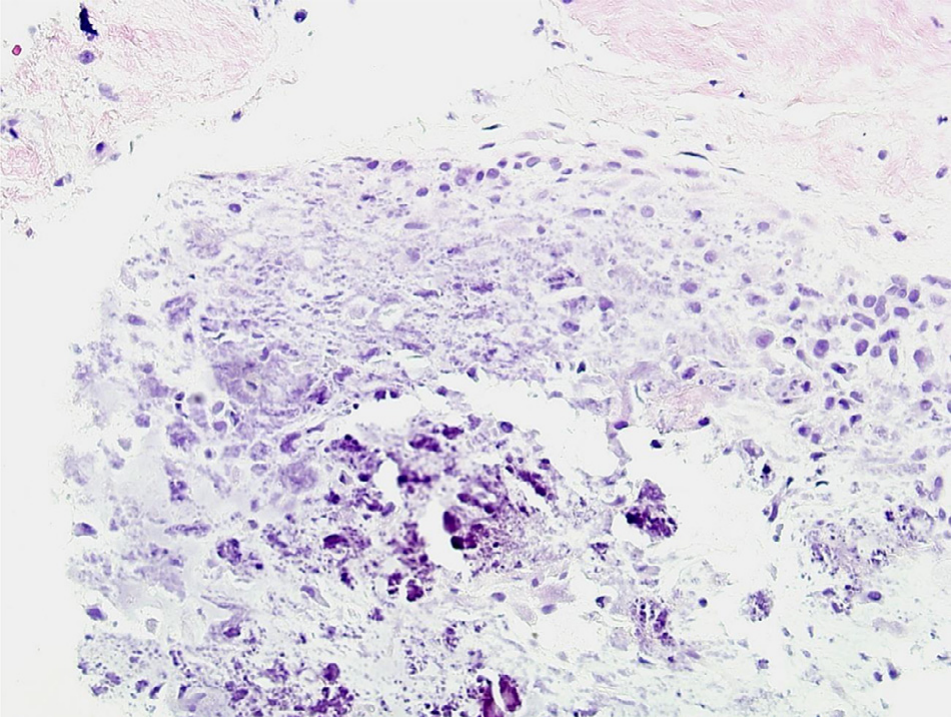
Fragment of tissue from core biopsy of the lesion. High magnification showing hypocellular connective tissue at the top of the image, and an extensively calcified chondromatous proliferation. Some of the cells in the right side of the image show mild to moderate atypia, but there is no necrosis, and the calcification has speckled pattern instead of "chicken wire" appearance. (HE, original magnification × 200).

**Fig 7:**
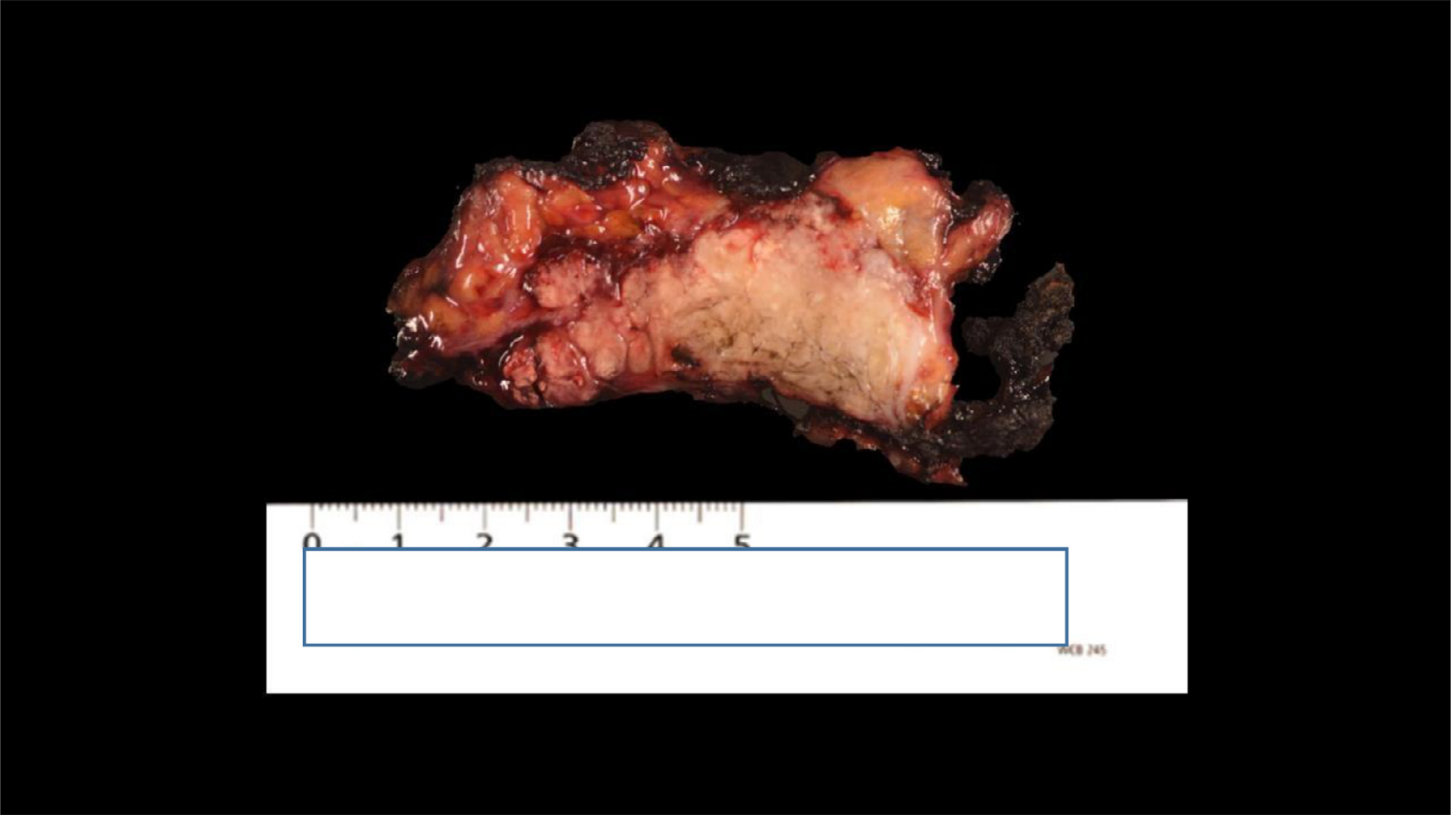
Macroscopic image of the lesion. It is a firm mass on palpation, surrounded by connective and adipose tissue in most of its outline. It appears poorly defined but not infiltrative in character and it comprises lobulated tissue in which peripheral yellow areas surround central spaces with a pearly white appearance.

**Fig 8:**
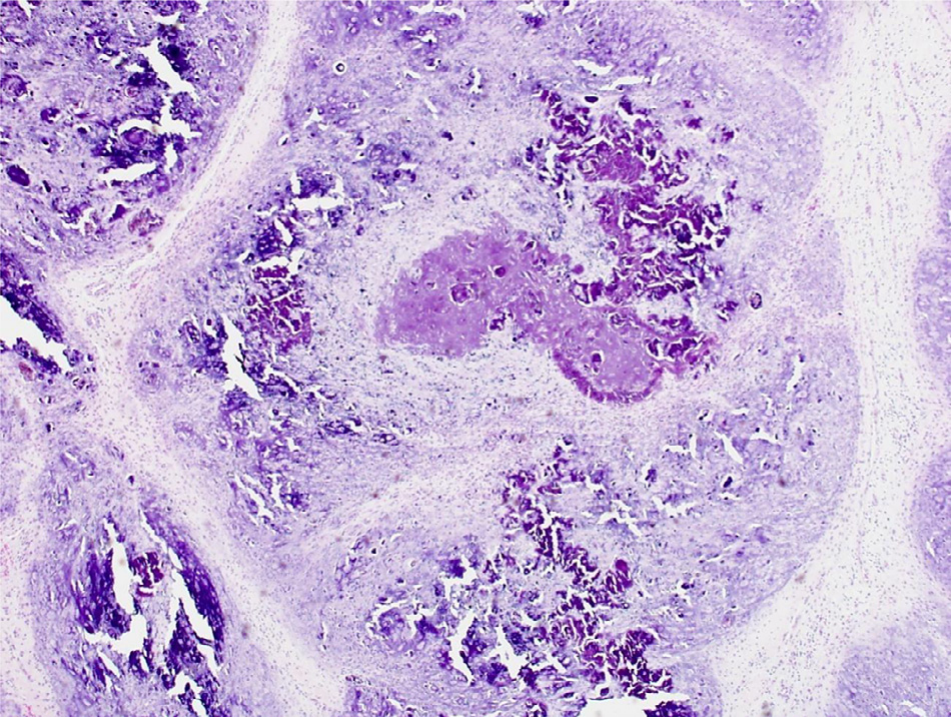
Image of the surgical excision specimen, showing one of the multiple nodules that characterize its microscopic appearance. The nodules are well circumscribed by a proliferation of bland spindle cells and are composed of metaplastic cartilage with characteristic speckled irregular calcifications in a concentric fashion. This is the hallmark of calcifying aponeurotic fibroma (HE, original magnification × 100).

The final diagnosis was reached after percutaneous and surgical biopsy, and subsequent cyto-histological analysis.

In conclusion, we present a rare case of a CAF with the lesion located in the soft tissue around the clavicle, an unusual location for this lesion. A thorough clinical evaluation is vital for accurate diagnoses; advanced radiographic and imaging evaluation can aid in preoperative planning, and a definitive diagnosis is obtained by histopathology. Surgical treatment and excision of the tumor produce favorable results; however, recurrence rates are high due to its multinodular characteristics, which may result in seeding or hourglass remnant tumor nests. Although it is a well-known entity, CAFs can be a diagnostic challenge, particularly when they occur at unusual ages and in atypical locations, as in our case. The characteristic images of CAFs may mimic those of malignant lesions and this should be considered in the differential diagnosis when chondroid and sarcomatous soft tissue lesions are suspected.

## Ethics approval

Publication approval was received from Netcare research operations committee. A copy of the approval is available for review by the Editor-in-Chief of this journal.

## Funding

No funding.

## Consent

Written informed consent was obtained from the patient for publication of this case report. A copy of the written consent is available for review by the editor-in-chief of this journal.

## Guarantor

Salvatore Marsico MD.

## Conflict of interest

None declared.
